# Association of Sleep Duration Ratio with a Reduced Risk of Heart Failure: Analysis of the 2017–2023 National Health and Nutrition Examination Survey

**DOI:** 10.3390/jcm15135047

**Published:** 2026-06-29

**Authors:** Narathorn Kulthamrongsri, Thanathip Suenghataiporn, Adivitch Sripusanapan, Smuch Siramongkholkarn, Thitiphan Srikulmontri, Chanokporn Puchongmart, Thanaboon Yinadsawaphan, Ben Thiravetyan, Kridhitach Ngarmukos, Koravich Lorlowhakarn, Nathasith Tangprasittichok, Abdulelah Nuqali, Ekamol Tantisattamo

**Affiliations:** 1 American Heart Association Comprehensive Hypertension Center at the University of California Irvine Medical Center, Division of Nephrology, Hypertension and Kidney Transplantation, Department of Medicine, University of California Irvine School of Medicine, Orange, CA 92868, USA; nkultham@uci.edu (N.K.); nutto233219@gmail.com (N.T.); 2 Internal Medicine Residency Program, University of Hawaii, Honolulu, HI 96813, USA; dayadivitch@gmail.com (A.S.); yinadsawaphan.thanaboon@mayo.edu (T.Y.); jamengarmukos@gmail.com (K.N.); 3 Department of Cardiovascular Medicine, Mayo Clinic, Phoenix, AZ 85054, USA; 4 Department of Internal Medicine, Griffin Hospital, Derby, CT 06418, USA; thanathip.sue@gmail.com; 5 Department of Medicine, Boston Medical Center Brighton, Boston, MA 02118, USA; s.siramongkholkarn@gmail.com (S.S.); rlowhakarn@gmail.com (K.L.); 6 Albert Einstein Medical Center, Philadelphia, PA 19141, USA; thitiphansrikulmontri@gmail.com; 7 Department of Internal Medicine, Texas Tech University Health Sciences Center, Lubbock, TX 79430, USA; chanokporn.puchongmart@ttuhsc.edu (C.P.); ben.thi@mahidol.edu (B.T.); 8 Phramongkutklao College of Medicine, Mahidol University, Bangkok 10400, Thailand; 9 Queen’s Heart Institute, The Queen’s Medical Center, Honolulu, HI 96813, USA; anuqali@queens.org; 10 Center for Global Cardiometabolic Health and Nutrition, University of California Irvine, Irvine, CA 92697, USA; 11 Multi-Organ Transplant Center, Section of Nephrology, Department of Internal Medicine, Corewell Health William Beaumont University Hospital, Oakland University William Beaumont School of Medicine, Royal Oak, MI 48073, USA; 12 Pacific Northwest University of Health Sciences, Yakima, WA 98901, USA; 13 Excellence Center for Organ Transplantation, Faculty of Medicine Ramathibodi Hospital, Mahidol University, Bangkok 10400, Thailand

**Keywords:** heart failure, sleep duration, weekend sleep recovery, NHANES, cardiovascular disease, public health

## Abstract

**Background/Objectives:** In the United States, HF prevalence is projected to progressively rise by 2030. Prior research suggests a strong association between reduced sleep duration and increased cardiovascular disease and HF risk. This study introduces an alternative parameter, the weekend sleep recovery (WSR), measured by the weekend-to-weekday sleep duration ratio (SDR), to evaluate its association with HF risk. **Methods:** We conducted a cross-sectional analysis of NHANES (National Health and Nutrition Examination Survey) 2017–2023 to examine self-reported sleep patterns. Participants were classified as WSR (SDR > 1) or non-WSR (SDR ≤ 1). Multivariate logistic regression assessed the association between WSR and HF, adjusting for demographics and comorbidities. **Results:** Among 8320 participants included in the fully adjusted analysis, WSR was associated with lower odds of HF compared with non-WSR (adjusted OR 0.73, 95% CI 0.55–0.96; *p* = 0.026). A significant interaction was observed between WSR and weekday sleep duration (P for interaction = 0.003), whereas no interaction was found with weekend sleep duration. In exploratory subgroup analyses, nominally significant associations were observed in several clinical subgroups; however, after correction for multiple comparisons, only participants without dyslipidemia retained statistical significance. No significant effect modification by race/ethnicity was observed (P for interaction = 0.436). **Conclusions:** Weekend sleep recovery was associated with lower odds of HF in this cross-sectional study. The association varied according to weekday sleep duration but was generally consistent across racial/ethnic groups. Given the observational design, these findings should be interpreted as associative rather than causal and warrant confirmation in prospective studies.

## 1. Introduction

In the United States, data from the National Health and Nutrition Examination Survey (NHANES) estimate the prevalence of heart failure (HF) to range from 1.9% to 2.6%, with projections indicating that the number of individuals affected may reach 8.5 million by 2030 [1,2,3].

Previous studies have shown that shorter sleep duration is associated with increased risk of cardiovascular disease (CVD) and HF. Sleeping fewer than 6 h per night has been associated with a 1.24-fold higher risk HF. Compared with a sleep duration of 7.0–7.9 h per night, individuals who sleep 6.0–6.9 h have a 1.29-fold higher risk. In contrast, long sleep duration has not been consistently associated with HF risk [4,5,6].

Weekend catch-up sleep (WCS), characterized by sleeping an additional two or more hours on weekends compared to weekdays, was associated with a reduced prevalence of CVD among individuals who usually sleep fewer than six hours on weekdays [7]. However, the association between variations in weekend and weekday sleep patterns and the risk of HF remains poorly understood.

Our study presents weekend sleep recovery (WSR) as an alternative parameter, defined using the weekend-to-weekday sleep duration ratio (SDR) instead of the absolute difference in sleep duration between weekdays and weekends. This approach aims to investigate the association between WSR and HF risk in a nationally representative sample, leveraging data from NHANES.

## 2. Methods

**Figure 1:**
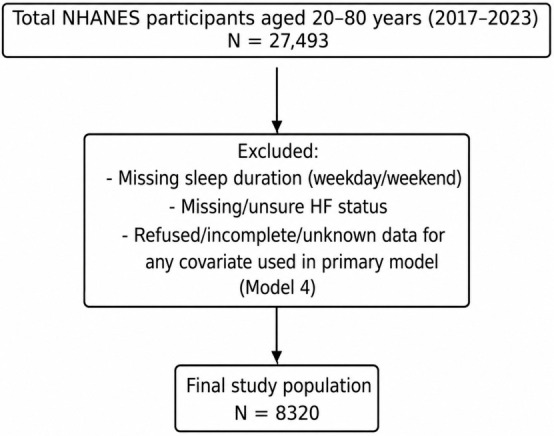
Flowchart of study participant selection from NHANES 2017–2023, showing inclusion and exclusion criteria leading to a final analytic sample of 8320 adults aged 20–80 years.

NHANES is a comprehensive and diverse series of surveys that assess general health, employment characteristics, nutritional status, lifestyle factors, and laboratory findings. Conducted by the Centers for Disease Control and Prevention (CDC) in collaboration with the National Center for Health Statistics (NCHS), this survey provides valuable insights into the health of the U.S. population. For this study, data from 27,493 adults aged 20 to 80 years, representing the civilian, non-institutionalized population between 2017 and 2023, were analyzed. Participants were included if they reported their sleep duration on both weekdays and weekends, along with their HF status. Individuals with missing data, unsure responses, or refusals were excluded, resulting in a final study population of 8320 participants (Figure 1).

This study did not necessitate consent or Institutional Review Board approval, as NHANES is a secondary dataset containing de-identified data that is publicly accessible.

### 2.1. Definition

#### 2.1.1. Sleep Duration

Participants reported their average nightly sleep duration for both weekdays and weekends. Sleep duration data were collected in NHANES through two questions: “How much sleep do you usually get at night on weekdays or workdays?” and “How much sleep do you usually get at night on weekends or non-workdays?” Responses ranged from 1 to 12, with 12 representing 12 or more hours of sleep. Participants who selected “don’t know” or “refused” to answer were excluded from the analysis.

#### 2.1.2. Weekend Sleep Recovery and Weekend-to-Weekday Sleep Duration Ratio

The “SDR” was calculated by dividing the reported sleep duration on weekends by the reported sleep duration on weekdays. Participants were classified into two groups based on their SDR: WSR and non-WSR, represented individuals with an SDR > 1 and ≤1, respectively.

#### 2.1.3. Cardiovascular Condition and Other Confounders

NHANES collects data on various self-reported health conditions through the general question, “Has a doctor or other health professional ever told you that you had [name of medical condition]?” Participants who reported a diagnosis of HF were classified as having the primary outcome of interest.

Potential confounders included demographic variables, namely race/ethnicity (RIDRETH3), sex (RIAGENDR), and age at screening (RIDAGEYR); social factors, including marital status (DMDMARTZ) and educational attainment (DMDEDUC2); and medical comorbidities, including obesity status based on body mass index (BMXBMI), smoking status (SMQ020), hypertension (BPQ020), diabetes mellitus (DIQ010), dyslipidemia (BPQ080), sleep-related variables comprising self-reported sleep disorder status (SLQ050), snoring frequency (SLQ030), and snorting/gasping frequency during sleep (SLQ040), as well as coronary artery disease (MCQ160C) and myocardial infarction (MCQ160E). Details of these variables are provided in Table 1 and Table 2.

### 2.2. Outcomes

The primary outcome was the association between WSR and self-reported HF, as defined by participants’ responses to the NHANES question asking whether a doctor or other health professional had ever told them they had HF. The analysis compared the odds of HF among individuals with WSR (sleep duration ratio [SDR] > 1) versus those without WSR (SDR ≤ 1). Multivariable logistic regression models (Models 1–5) were used to estimate these associations, with sequential adjustment for potential confounding variables as described in the Statistical Analysis section.

Secondary analyses evaluated the association between WSR and HF across subgroups stratified as described in the Statistical Analysis section. These subgroup analyses were exploratory and hypothesis-generating.

### 2.3. Statistical Analysis

Categorical data were presented as proportion, while continuous variables were expressed as means with standard deviations or median and interquartile range (IQR). Baseline differences for categorical variables were evaluated using the Chi-square test, and continuous variables were compared using Student’s *t*-test. Descriptive analyses used all available data for each variable, whereas multivariable logistic regression analyses were restricted to participants with complete data for all covariates included in each adjusted model.

Logistic regression models were applied to evaluate the associations between WSR and HF as follows:

Model 1 (Unadjusted): Univariate logistic regression

Model 2 (Demographic-adjusted): Model 1 + demographic variables

Model 3 (Social factor–adjusted): Model 2 + social factor variables

Model 4 (Medical comorbidity–adjusted) (Primary adjusted model): Model 3 + medical comorbidity and sleep-related variables

Model 5 (CAD/MI-adjusted): Model 4 + CAD and MI

CAD and MI were not included in the primary adjusted model (Model 4) because they are highly correlated with HF and several other covariates, raising concerns about multicollinearity. Additionally, CAD and MI may lie on the causal pathway between sleep pattern variability and HF, functioning partly as mediators rather than pure confounders. Therefore, they were incorporated as additional covariates only in Model 5 and in sensitivity analyses to assess the robustness of the associations.

Sensitivity analyses examined the association between WSR and HF across subgroups defined by age (<65 vs. ≥65 years), sex, smoking status, obesity (BMI < 30 vs. ≥30 kg/m^2^), diabetes mellitus, hypertension, dyslipidemia, coronary artery disease (CAD), and myocardial infarction (MI). Effect modification by race/ethnicity was evaluated using interaction analyses. Interactions between WSR and weekday and weekend sleep duration were also assessed. E-values were calculated to assess the robustness of the observed association to potential unmeasured confounding.

Results are presented as odds ratios (ORs) with 95% confidence intervals (CIs). All *p*-values were two-sided. Subgroup analyses were considered exploratory, and a Bonferroni-corrected significance threshold of *p* < 0.025 was applied to prespecified binary subgroup analyses. All statistical analyses were performed using Stata version 15.2.

## 3. Results

### 3.1. Baseline Characteristics of the Study Population

A total of 27,493 NHANES participants provided responses regarding their HF status, while 17,519 and 17,510 completed the sleep survey for weekdays and weekends, respectively. After applying the exclusion criteria, the final study cohort for the primary model (Model 4) comprised 8320 adults with available data on SDR, HF status, and all covariates. The overall mean sleep duration was 7.7 ± 1.7 h on weekdays and 8.3 ± 1.8 h on weekends. Significant differences were observed between the non-HF and HF groups in weekday sleep and sleep duration ratios, but not in weekend sleep durations. Weekday sleep duration was significantly shorter in participants with HF compared to non-HF counterparts (mean difference: −0.26, 95% CI: −0.43 to −0.10, *p* = 0.002) (Table 1).

Participants in the non-WSR group (SDR ≤ 1) were older (mean age 55.5 ± 18.6 years) compared to those in the WSR group (SDR > 1; mean age 34.1 ± 22.5 years). The non-WSR group also had higher proportions of individuals with hypertension, diabetes mellitus, and dyslipidemia, reflecting important clinical differences between the groups (Table 2).

**Figure 2:**
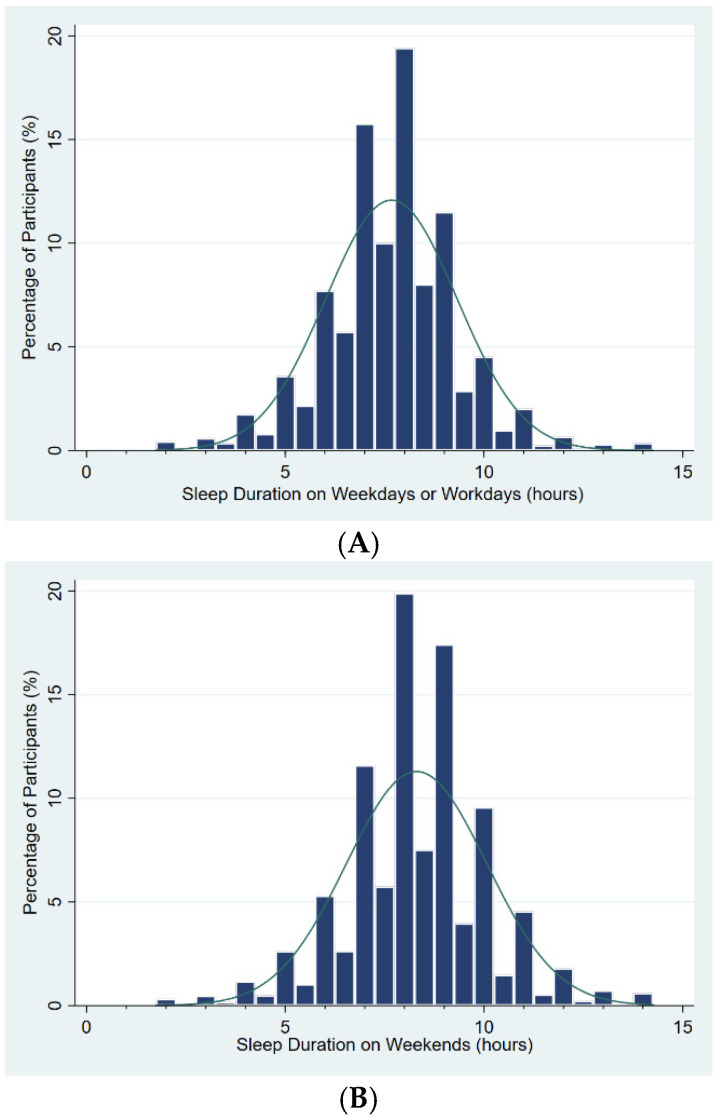

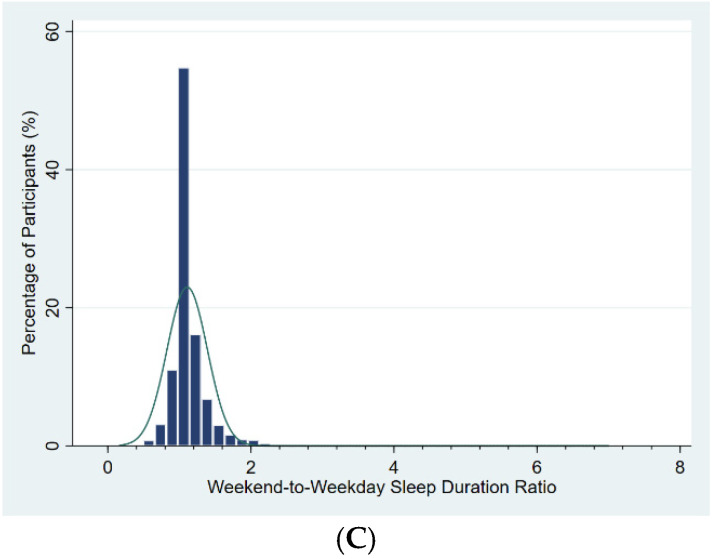
Distribution of sleep duration variables in the study population. (**A**) Histogram of self-reported sleep duration on weekdays or workdays (hours). (**B**) Histogram of self-reported sleep duration on weekends (hours). (**C**) Histogram of the weekend-to-weekday sleep duration ratio. Bars represent the percentage of participants within each interval, and the overlaid curves indicate kernel density estimates. Most participants reported approximately 7–8 h of sleep per night, with weekend-to-weekday sleep duration ratios clustering around 1.0, indicating similar sleep duration across weekdays and weekends.

**Figure 3:**
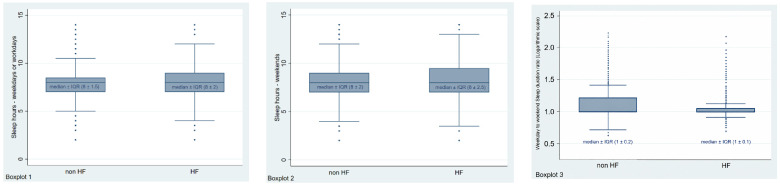
Boxplots comparing sleep patterns between individuals with heart failure (HF) and without heart failure (non-HF). Boxplot 1 shows weekday/workday sleep hours, Boxplot 2 shows weekend sleep hours, and Boxplot 3 shows the ratio of weekend-to-weekday sleep duration, after trimming the upper and lower 1% of the SDR distribution and applying a logarithmic scale to the *Y*-axis. Each plot displays the distribution, median, interquartile range, and outliers for each group.

The sleep duration patterns of the study population exhibited a normal distribution for both weekday sleep duration in hours and the weekend-to-weekday SDR, as illustrated in the histograms (Figure 2A,B). The line plot (Figure 2C) demonstrates considerable variability in sleep durations, ranging from 0 to approximately 15 h for both weekdays and weekends. The sleep patterns of individuals with and without HF were compared using boxplots, which illustrate weekday and weekend sleep duration, as well as the weekend-to-weekday sleep ratio, showing the distribution, median, interquartile range, and outliers for each group (Figure 3).

### 3.2. Primary and Secondary Outcome

Weekend sleep recovery (WSR), defined as a sleep duration ratio (SDR) > 1, was associated with substantially lower odds of prevalent HF in the unadjusted analysis (OR 0.47, 95% CI 0.40–0.56; *p* < 0.001). After adjustment for demographic characteristics including age, race, and sex, the association was attenuated and no longer statistically significant (OR 0.86, 95% CI 0.72–1.02; *p =* 0.091). Additional adjustment for marital status and educational level yielded similar results (OR 0.85, 95% CI 0.71–1.02; *p =* 0.084).

Following further adjustment for body mass index, smoking status, hypertension, diabetes mellitus, dyslipidemia, and sleep-related variables, WSR was independently associated with lower odds of HF (OR 0.73, 95% CI 0.55–0.96; *p =* 0.026). However, after additional adjustment for concurrent ischemic heart diseases, including coronary artery disease and myocardial infarction, the association was attenuated and became borderline significant (OR 0.75, 95% CI 0.55–1.01; *p =* 0.057) (Table 3).

### 3.3. Sensitivity Analyses

#### 3.3.1. Weekday and Weekend Sleep Duration Interaction Analyses

**Figure 4:**
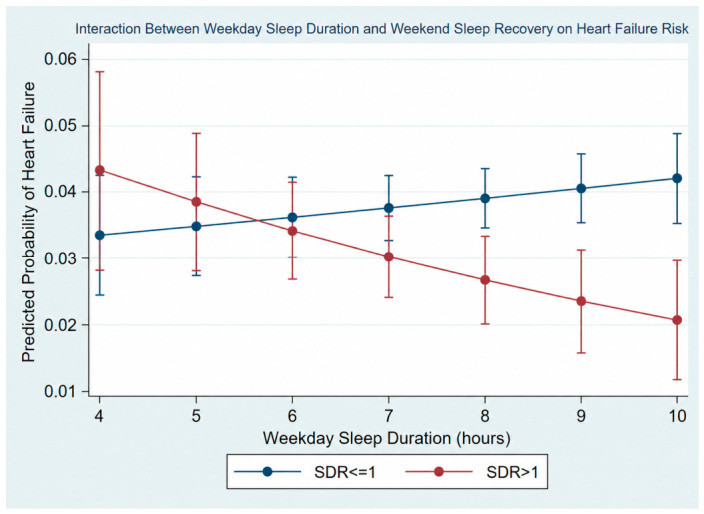
Predicted probability of heart failure according to weekday sleep duration and weekend sleep recovery status.

In Model 4, participants with an SDR >1 had lower odds of HF than those with an SDR ≤ 1 (OR 0.73, 95% CI 0.55–0.96, *p =* 0.026). Sensitivity analysis yielded an E-value of 2.08 for the point estimate and 1.25 for the confidence limit closest to the null. In analyses examining effect modification, a significant interaction was observed between SDR and weekday sleep duration (interaction OR 0.79, 95% CI 0.67–0.92, *p =* 0.003). Among participants with an SDR ≤ 1, each additional hour of weekday sleep was associated with a nonsignificant increase in the odds of HF (OR 1.07, 95% CI 0.99–1.15), whereas among those with an SDR > 1, the corresponding combined association was inverse (combined OR ≈ 0.84). In contrast, no significant interaction was observed between SDR and weekend sleep duration (interaction OR 0.91, 95% CI 0.78–1.06, *p =* 0.217), with combined estimates indicating little association between weekend sleep duration and HF among participants with an SDR >1 (combined OR ≈ 0.98). These findings suggest that the association between sleep duration and HF may vary according to weekday sleep patterns and the relative balance between weekend and weekday sleep (Table 4, Figure 4).

#### 3.3.2. Comorbidities Subgroup Analyses

**Figure 5:**
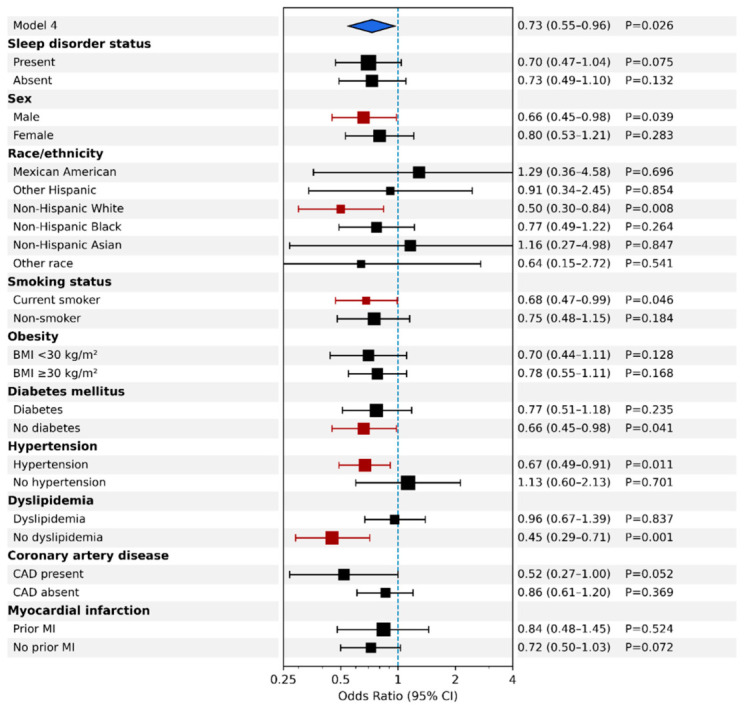
Association between weekend sleep recovery and heart failure across clinically relevant subgroups. Forest plot showing odds ratios (ORs) and 95% confidence intervals (CIs) for the association between weekend sleep recovery (sleep duration ratio > 1) and prevalent heart failure. Squares represent point estimates, with area proportional to the inverse variance of each estimate. Horizontal lines denote 95% confidence intervals. The blue diamond indicates the overall adjusted effect estimate from Model 4. Dark red squares indicate nominally significant associations (*p* < 0.05). The vertical dashed line represents no association (OR = 1.0).

In subgroup analyses, nominally significant inverse associations between WSR and HF were observed among men (OR 0.66, 95% CI 0.45–0.98, *p =* 0.039), current smokers (OR 0.68, 95% CI 0.47–0.99, *p =* 0.046), participants without diabetes mellitus (OR 0.66, 95% CI 0.45–0.98, *p =* 0.041), those with hypertension (OR 0.67, 95% CI 0.49–0.91, *p =* 0.011), and individuals without dyslipidemia (OR 0.45, 95% CI 0.29–0.71, *p =* 0.001). However, after Bonferroni correction (*p* < 0.025), the association among participants without dyslipidemia remained statistically significant. No significant interaction by race/ethnicity was observed (P for interaction = 0.436), suggesting broadly consistent associations across racial/ethnic groups (Table 5, Figure 5).

Adjusted predicted probabilities of heart failure according to weekday sleep duration among adults aged ≥ 20 years, stratified by weekend sleep recovery (WSR) status. Predicted probabilities were estimated from a multivariable logistic regression model including an interaction term between weekday sleep duration and WSR status and adjusting for demographic characteristics, socioeconomic factors, body mass index, smoking status, hypertension, diabetes, sleep-related variables, and comorbid conditions. Error bars indicate 95% confidence intervals. WSR was defined as a weekend-to-weekday sleep duration ratio > 1, whereas no WSR was defined as a ratio ≤ 1.

## 4. Discussion

This study observed an inverse association between WSR and HF, with individuals reporting weekend sleep recovery having lower odds of HF. The direction and magnitude of the association were generally similar across regression models. Given the cross-sectional design, these findings should be interpreted as associative rather than indicative of a protective effect. Reverse causality also represents an important alternative explanation. Individuals with established HF may experience altered sleep patterns due to fatigue, reduced functional capacity, or sleep disruption from symptoms such as nocturnal dyspnea and orthopnea. Consequently, the observed SDR may reflect disease-related changes in sleep behavior rather than a determinant of HF. In addition, obstructive sleep apnea (OSA), an established risk factor for cardiovascular disease and HF, may influence sleep architecture and duration and thereby confound the observed association. Although adjustment for self-reported sleep disorder status, snoring frequency, and snorting/gasping frequency yielded similar results, residual confounding from undiagnosed sleep-disordered breathing (SDB) cannot be excluded.

Subgroup analyses suggested that the inverse association between WSR and HF may vary across demographic and clinical characteristics. However, given the multiple comparisons performed, findings from binary subgroup analyses should be interpreted cautiously. Additionally, no significant interaction by race/ethnicity was observed (P for interaction = 0.436), suggesting broadly consistent associations across racial/ethnic groups. However, limited sample sizes in some subgroups reduced estimate precision and may limit generalizability to specific populations. The stronger association observed in men is noteworthy, as sex-specific differences in OSA prevalence, symptom presentation, HF phenotypes [8] and cardiovascular consequences are well recognized and may influence the observed associations. However, because NHANES self-reported HF does not distinguish between HF with preserved (HFpEF) and reduced ejection fraction (HFrEF), subtype-specific differences could not be evaluated. Men are more likely to exhibit classic OSA symptoms and experience greater adverse cardiovascular sequelae, whereas women often present with atypical OSA symptoms, potentially leading to underrecognition and differences in disease burden. In addition, HF phenotypes differ by sex, with HFrEF occurring more commonly in men and HFpEF being more prevalent in women [9]. These biological and clinical differences may partly explain the heterogeneity observed across sex strata. Nevertheless, subgroup findings should be considered exploratory, and future studies incorporating objective assessments of SDB are needed to clarify whether sex-related differences in OSA, other sleep characteristics and HF phenotype modify the relationship between WSR and HF.

Our study builds on prior work introducing WSR as a relative measure of compensatory sleep. Traditional WCS defines recovery as an absolute ≥ 2 h difference between weekday and weekend sleep, which may overlook smaller but proportionally meaningful changes (e.g., 4 vs. 5 h). By using SDR, we capture proportional sleep compensation, allowing assessment of associations even when weekend extension is less than two hours. This approach may better reflect real-world sleep patterns, particularly for individuals unable to achieve large absolute increases in weekend sleep. Although individuals with markedly different absolute sleep durations may share identical SDR values, stratified and interaction analyses suggested that the association between WSR and HF varied according to weekday sleep duration but not weekend sleep duration. These findings imply that the relative balance between weekday and weekend sleep may be more informative than weekend sleep duration alone. Nevertheless, the clinical validity and optimal definition of SDR warrant further study.

Our results aligned with previous research show that both short and long sleep durations are linked to an increased risk of HF and other cardiovascular conditions [6,8]. Prior studies have demonstrated U-shaped associations between sleep duration and HF risk, emphasizing the importance of optimal sleep for cardiovascular health [6]. Evidence also suggests that insufficient sleep is associated with increased inflammation, dysregulated appetite hormones, and higher BMI, which are well-established risk factors for HF [10]. Potential mechanisms linking sleep patterns and HF also include autonomic and neurohumoral dysregulation and metabolic dysfunction. SDB, particularly obstructive sleep apnea, may further contribute through intermittent hypoxia and sleep fragmentation, with sex-related differences potentially influencing these associations [10]. Moreover, research on WCS highlights its potential to reduce CVD prevalence, although its direct impact on HF remains uncertain [7]. Notably, a recent NHANES 2017–2023 analysis by Zhang et al. [11] reported that WCS was associated with lower odds of hypertension, which is one of the HF-related comorbidities [12]. Nevertheless, these prior studies, together with our findings, are observational and do not establish causality. Whether recovery sleep itself contributes to cardiovascular health or instead reflects underlying differences in health status and disease burden remains uncertain. Longitudinal studies are required to clarify the temporal relationship between WSR and HF and to determine whether WSR represents a marker or a potential modifiable factor in the pathway leading to HF.

### Limitation

This study has several limitations. First, residual confounding cannot be excluded, including factors such as underreported alcohol use, social determinants of health, undiagnosed SDB, and HF-related medications that may influence sleep quality and duration. In addition, NHANES lacks objective measures of OSA severity and does not reliably capture medication indication or adherence. Furthermore, self-reported HF does not distinguish between HF phenotypes, precluding assessment of subtype-specific associations and limiting interpretation of sex-related differences [10]. Second, while SDR has its advantages, it also has a key limitation: it does not distinguish between individuals with different sleep patterns but identical SDR values. For instance, someone sleeping 3 h on weekdays and 4.5 h on weekends has the same SDR of 1.5 as someone sleeping 6 h on weekdays and 9 h on weekends. Third, the cross-sectional design of NHANES precludes causal inference. Missing data and reliance on self-reported measures may also introduce recall, misclassification or social desirability bias. Although marked baseline differences existed between the WSR and non-WSR groups, with individuals without WSR generally being older, more likely to smoke, and having a greater burden of comorbidities, E-value analysis suggested that a moderately strong unmeasured confounder would be required to fully explain the observed association. Finally, the findings are based on U.S. population data, which may limit generalizability to other populations.

## 5. Conclusions

Our findings suggest that WSR is associated with lower odds of HF, particularly among individuals with intermediate weekday sleep durations and certain cardiometabolic subgroups. These observations may indicate a relationship between recovery sleep patterns and cardiovascular risk; however, prospective and mechanistic studies are required before drawing conclusions regarding causality, clinical implications, or sleep recommendations. Reverse causality has not been excluded.

## Figures and Tables

**Table 1:** Baseline characteristics of individuals categorized as HF vs. non-HF.

Variables	Non-HF	HF	Mean Difference	*p*-Value
How much sleep do you get during weekdays? (hours)	7.6 ± 1.6 (8 +/− 1.5) (*n* = 16,111)	7.9 ± 2.2 (8 +/− 2) (*n* = 681)	−0.3 ± 0.1 (−0.43, −0.10)	<0.01
How much sleep do you get during weekends? (hours)	8.3 ± 1.7 (8 +/− 2) (*n* = 16,104)	8.2 ± 2.2 (8 +/− 2.5) (*n* = 679)	0.0 ± 0.1 (−0.12, 0.21)	0.62
Weekday-to-weekend sleep duration ratio	1.1 ± 0.3 (1 +/− 0.2) (*n* = 16,052)	1.1 ± 0.3 (1 +/− 0.1) (*n* = 676)	0.04 ± 0.0 (0.02, 0.07)	<0.01

Baseline sleep characteristics (mean ± SD (standard deviation) or median +/− interquartile range (IQR) or *n* [%]) of individuals categorized as heart failure (HF) vs. non-HF. This table summarizes weekday sleep duration, weekend sleep duration, and the weekday-to-weekend sleep duration ratio for individuals with and without HF. Significant group differences were observed in weekday sleep duration and sleep duration ratios, whereas weekend sleep duration did not differ meaningfully between groups. Sample sizes shown for individual sleep variables reflect the number of participants with available responses to the corresponding questionnaire items. The primary analytic sample (model 4) for multivariable analyses (*n* = 8320) was restricted to participants with complete data for all covariates included in the fully adjusted model.

**Table 2:** Baseline characteristics of individuals categorized as Non-WSR (SDR ≤ 1) vs. WSR (SDR > 1).

Variables	Non-WSR (SDR ≤ 1) (*n* = 9691)	WSR (SDR > 1) (*n* = 17,802)	*p*-Value
Age (years)	55.5 ± 18.6	34.1 ± 22.5	<0.0001
Women (%)	5045 (52.1%)	6059 (52.9%)	0.233
White (%)	4869 (50.2%)	4555 (39.8%)	<0.0001
Body mass index (BMI)	29.7 ± 7.3	30.3 ± 8.5	<0.0001
Overweight status (%)	1938 (38.7%)	1679 (39.9%)	0.356
Active smoker (%)	4290 (44.7%)	2796 (36.0%)	<0.0001
Marital Status (married) (%)	5093 (54.0%)	4313 (57.0%)	<0.0001
Education: above college (%)	2640 (28.0%)	2240 (29.6%)	0.098
Heart failure (%)	504 (5.4%)	198 (2.6%)	<0.0001
Coronary artery disease (%)	632 (6.7%)	190 (2.5%)	<0.0001
Myocardial Infarction (%)	554 (5.9)	206 (2.7)	<0.0001
Hypertension (%)	4111 (42.4%)	2412 (30.1%)	<0.0001
Diabetes mellitus (%)	1581 (16.3%)	914 (8.1%)	<0.0001
Dyslipidemia (%)	3953 (40.8%)	2397 (29.9%)	<0.0001
Sleep disorder (%)	1526 (30.5%)	1070 (25.4%)	<0.0001
How much sleep do you get during weekdays? (hours)	8.1 ± 1.7	7.2 ± 1.5	<0.0001
How much sleep do you get during weekends? (hours)	7.7 ± 1.7	9.0 ± 1.6	<0.0001
Weekday-to-weekend sleep duration ratio	1.0 ± 0.1	1.3 ± 0.3	<0.0001

Baseline characteristics (mean ± SD (standard deviation)) of individuals categorized as Non-WSR (weekday-to-weekend sleep duration ratio, SDR ≤ 1) and WSR (SDR > 1). Significant differences were observed between the two groups in terms of age, race, BMI, smoking status, marital status, and several health conditions, including heart failure, coronary artery disease, myocardial infarction, hypertension, diabetes mellitus, and dyslipidemia. Sleep duration patterns, including weekday and weekend sleep durations and sleep duration ratio, also differed significantly between the groups. Sample sizes shown for individual sleep variables reflect the number of participants with available responses to the corresponding questionnaire items. The primary analytic sample (model 4) for multivariable analyses (*n* = 8320) was restricted to participants with complete data for all covariates included in the fully adjusted model.

**Table 3:** Association between weekend sleep recovery (WSR; sleep duration ratio > 1) and heart failure across sequential logistic regression models.

Model	Covariates Included	*N*	OR (95% CI) for WSR (SDR > 1)	*p*-Value
Model 1	Unadjusted	16,985	0.47 (0.40–0.56)	<0.001
Model 2	Age, race, sex	16,985	0.86 (0.72–1.02)	0.091
Model 3	Model 2 + marital status, education	16,931	0.85 (0.71–1.02)	0.084
Model 4 (Primary)	Model 3 + BMI, smoking, hypertension, diabetes, dyslipidemia, sleep-related variables	8320	0.73 (0.55–0.96)	0.026
Model 5	Model 4 + coronary artery disease and myocardial infarction	8320	0.75 (0.55–1.01)	0.057

Abbreviations: OR, odds ratio; CI, confidence interval; WSR, weekend sleep recovery; SDR, sleep duration ratio; BMI, body mass index. Association between weekend sleep recovery (WSR; sleep duration ratio >1) and heart failure (HF) across unadjusted and adjusted models. Model 1 presents the unadjusted association. Model 2 adjusts for demographic variables (age, race, and gender). Model 3 further adjusts for social factors (marital status and education level). Model 4 additionally adjusts for medical comorbidities (body mass index, smoking status, hypertension status, diabetes mellitus status, dyslipidemia status, and sleep-related variables). Model 5 additionally adjusts for concurrent heart diseases (coronary artery disease and myocardial infarction).

**Table 4:** Interaction analyses of weekday and weekend sleep duration with sleep duration ratio and odds of heart failure.

Variable	OR	95% CI	*p*-Value
Model 4 * (Primary model without interaction)			
SDR >1 vs. SDR ≤1	0.73	0.55–0.96	0.026
Weekday sleep duration interaction model * (SLD012)			
SDR > 1	3.97	1.24–12.71	0.020
Weekday sleep duration (per hour)	1.07	0.99–1.15	0.083
SDR × weekday sleep duration	**0.79**	**0.67–0.92**	**0.003**
Weekend sleep duration interaction model * (SLD013)			
SDR > 1	1.49	0.39–5.72	0.560
Weekend sleep duration (per hour)	1.08	1.00–1.16	0.062
SDR × weekend sleep duration	0.91	0.78–1.06	0.217

* Adjusted for race/ethnicity, sex, age, marital status, education level, body mass index, smoking status, hypertension, diabetes, hyperlipidemia, physician-diagnosed sleep disorder, frequency of trouble falling asleep, and frequency of waking during the night. Values are presented as odds ratios (ORs) with 95% confidence intervals (CIs).

**Table 5:** Subgroup analyses of the association between weekend sleep recovery (SDR > 1) and heart failure.

**Main Analysis**	**OR (95% CI)**	***p*** **-Value**
**Model 4**	0.73 (0.55–0.96)	0.026
**Subgroup**	**OR (95% CI)**	***p*** **-Value**
**Sleep disorder status**		
Present	0.70 (0.47–1.04)	0.075
Absent	0.73 (0.49–1.10)	0.132
**Sex**		
Male	0.66 (0.45–0.98)	0.039
Female	0.80 (0.53–1.21)	0.283
**Race/ethnicity**		0.436 ^
Mexican American	1.29 (0.36–4.58)	0.696
Other Hispanic	0.91 (0.34–2.45)	0.854
Non-Hispanic White	0.50 (0.30–0.84)	0.008
Non-Hispanic Black	0.77 (0.49–1.22)	0.264
Non-Hispanic Asian	1.16 (0.27–4.98)	0.847
Other race	0.64 (0.15–2.72)	0.541
**Smoking status**		
Current smoker	0.68 (0.47–0.99)	0.046
Non-smoker	0.75 (0.48–1.15)	0.184
**Obesity**		
BMI <30 kg/m^2^	0.70 (0.44–1.11)	0.128
BMI ≥30 kg/m^2^	0.78 (0.55–1.11)	0.168
**Diabetes mellitus**		
Diabetes	0.77 (0.51–1.18)	0.235
No diabetes	0.66 (0.45–0.98)	0.041
**Hypertension**		
Hypertension	0.67 (0.49–0.91)	0.011
No hypertension	1.13 (0.60–2.13)	0.701
**Dyslipidemia**		
Dyslipidemia	0.96 (0.67–1.39)	0.837
No dyslipidemia	0.45 (0.29–0.71)	0.001
**Coronary artery disease**		
CAD present	0.52 (0.27–1.00)	0.052
CAD absent	0.86 (0.61–1.20)	0.369
**Myocardial infarction**		
Prior MI	0.84 (0.48–1.45)	0.524
No prior MI	0.72 (0.50–1.03)	0.072

**Association between weekend sleep recovery (WSR; sleep duration ratio >1) and heart failure (HF): main analysis and subgroup analyses using the fully adjusted model (Model 4).** Model 4 adjusted for demographic variables (age, sex, and race/ethnicity), socioeconomic factors (marital status and educational attainment), body mass index (BMI), smoking status, hypertension, diabetes mellitus, dyslipidemia, sleep disorder status, and sleep-related variables including weekday sleep duration and sleep latency. Subgroup analyses were performed according to sleep disorder status, sex, race/ethnicity, smoking status, obesity, diabetes mellitus, hypertension, dyslipidemia, coronary artery disease (CAD), and prior myocardial infarction (MI). Odds ratios (ORs) and 95% confidence intervals (CIs) represent the association between WSR (sleep duration ratio >1) and prevalent heart failure, with participants having a sleep duration ratio ≤ 1 serving as the reference group. **^** P-interaction was calculated from multiplicative interaction terms between WSR and the corresponding subgroup variable in the primary adjusted model 4. **Abbreviations:** BMI, body mass index; CAD, coronary artery disease; CI, confidence interval; HF, heart failure; MI, myocardial infarction; OR, odds ratio; WSR, weekend sleep recovery.

## Data Availability

The data analyzed in this study were obtained from the publicly available National Health and Nutrition Examination Survey (NHANES) database (https://www.cdc.gov/nchs/nhanes/ (accessed on 1 November 2024)). Additional processed data and analysis outputs generated during the current study are not publicly available but are available from the corresponding author upon reasonable request.
